# Animals, anoxic environments, and reasons to go deep

**DOI:** 10.1186/s12915-016-0266-1

**Published:** 2016-06-07

**Authors:** Marek Mentel, Aloysius G. M. Tielens, William F. Martin

**Affiliations:** Department of Biochemistry, Faculty of Natural Sciences, Comenius University, Mlynská dolina CH-1, 842 15 Bratislava, Slovakia; Faculty of Veterinary Medicine, Department of Biochemistry and Cell Biology, Utrecht University, Utrecht, The Netherlands; Department of Medical Microbiology and Infectious Diseases, Erasmus University Medical Center, Rotterdam, The Netherlands; Institute of Molecular Evolution, University of Düsseldorf, 40225 Düsseldorf, Germany

## Abstract

One of the classic questions in the early evolution of eukaryotic life concerns the role of oxygen. Many unicellular eukaryotes are strict anaerobes and many animals have long anoxic phases in their life cycle. But are there also animals that can complete their life cycle without oxygen? In an ongoing debate in *BMC Biology*, Danovaro and colleagues say “yes” while Bernhard and colleagues say “no”. The debate concerns reports of anoxic metazoans in deep sea anaerobic habitats.

In a Correspondence contribution to *BMC Biology*, Danovaro and colleagues [1] weigh in to defend the view that the loriciferans they reported in 2010 from anoxic sediments beneath the Mediterranean brine were living at the time of collection [2]. That view has recently been challenged by Bernhard et al. [3], who say that the loriciferans in question were not alive, but were dead and merely well preserved. The issue at hand is whether metazoans exist that can complete their entire life cycles without oxygen. This is important, but what is at stake?

## Anaerobic eukaryotes—common and widespread

Why is the issue of whether animals can complete a life cycle without oxygen so interesting? It has to do with the ecology of our own evolutionary past. The evolutionary significance of eukaryotic anaerobes has changed profoundly during the past two decades and this process of change is still very much in progress. In the mid 1980s, when the ribosomal RNA tree of life dominated views about eukaryote evolution, eukaryotic anaerobes were seen as primitive oddballs in the tree of eukaryotic life: unicellular, “early-diverging” ancestors of oxygen-breathing eukaryotes—the ones like us that have typical O_2_-respiring mitochondria. But the more closely people investigated eukaryotic anaerobes, the more it became apparent that they all had organelles of mitochondrial origin (OMO) after all, but anaerobic ones that function without oxygen [4]. The first members in the family of anaerobic mitochondria to be described were hydrogenosomes. Discovered by Miklos Müller in 1973 in a group of unicellular eukaryotes called trichomonads, hydrogenosomes generate ATP via H_2_-producing fermentation and are now known from diverse eukaryotic anaerobes (reviewed in [4]). The next new members in the mitochondrial family were mitosomes, which were first reported in the intestinal parasite *Entamoeba histolytica* [5] and characterized in the human parasite *Trachipleistophora hominis* [6]. Then came the report of true intermediates between mitochondria and hydrogenosomes: the hydrogen-producing mitochondria of a ciliate [7]. Together with the anaerobic mitochondria of parasitic metazoans that produce propionate and acetate as end products of energy metabolism [4], these discoveries revealed that organelles of mitochondrial origin represent a full spectrum of physiological forms.

At the same time, views of eukaryotic phylogeny changed such that the eukaryotic anaerobes, rather than branching deeply, were found to occur throughout all branches of the eukaryotic tree. Owing to the circumstance that anaerobes from diverse branches of the eukaryotic tree, including algae, all use subsets of the same handful of enzymes, there can be little doubt—from the standpoint of physiology—that the eukaryote common ancestor was a facultative anaerobe, the mitochondria of which were well-suited to ATP synthesis with or without oxygen [4]. It follows that aerobic, anaerobic, and facultatively anaerobic eukaryotes arose through ecological specialization towards their respective niches. That view of a facultatively anaerobic eukaryote ancestor fits well with current views on oxygen in Earth history, which have it that oxygen levels in the oceans were extremely low up until about 600 million years ago, or maybe even into the Paleozoic era [8]. The first eukaryotes and the first animals were marine inhabitants. It follows, therefore, that eukaryotes arose and diversified in environments where O_2_ had very low availability [4]. So that brings us to the big question.

## Regarding oxygen in animals, how low can you go?

This is where Danovaro et al. [1] and Bernhard et al. [3] go head to head. The story starts with the report by Danovaro et al. [2] of animals—three new species of the phylum Loricifera isolated in the permanently anoxic sediments sampled from the deep anoxic hypersaline basin of the Mediterranean Sea, L’Atalante basin. Their conclusion was that the animals live there permanently and, hence, complete their entire life cycle in the absence of oxygen. But that conclusion was challenged recently by Bernhard et al. [3], who questioned whether the newly found loriciferans were actually alive at the time of sampling, offering the alternative explanation that the animals in question in Danovaro et al. [2] were well preserved in the anoxic brine but dead [3]. This prompted Danovaro et al. [1] to reply, reinforcing their prior conclusions while taking into account (i) cell and tissue staining, (ii) incorporation of radiolabelled substrates, (iii) CellTracker Green labelling, and (iv) molecular analyses. In addition, they cite further evidence based on (a) the presence of intact loriciferans in different layers of sediments beneath the brine, (b) the presence of nearly all Loricifera life cycle stages in the same anoxic basin (L’Atalante basin of the Mediterranean), (c) the high abundance of Loricifera per unit of the sediment compared with other investigated sites worldwide, and finally (d) species richness and species distribution in anoxic conditions, which allowed them to back their initial conclusion. They counter that Bernhard et al. [3] experienced technical difficulties during sampling operations such that they could not get samples of permanently anoxic sediments from beneath the deep-sea hypersaline brines from where Danovaro et al. [2] recovered their samples. So it appears to be a stalemate at present; or is it a showdown at high noon?

## Wanted: loriciferans, dead or alive

The current debate should focus interest not solely on the old Wild West dead-or-alive issue but also on the rich biology in these habitats and the importance of obtaining new samples from the sediments in question and similar habitats. Indeed, there is no debate about the ability of unicellular eukaryotes to survive in the anoxic brine, nor is there debate about animals living on the margins of the anoxic zone [3]. The issue is the ability of metazoans (multicellular eukaryotes) to survive in the strictly anaeorbic zone. Ideally, one would like to see some evidence for actively transcribed genes in loriciferans from these habitats. That would also tell us a lot about how they are growing with respect to core carbon and energy metabolism. In particular, one would want to know whether these animals harbor and express any of the genes that protists use to survive in anaerobic environments, such as [FeFe]-hydrogenase, pyruvate:ferredoxin oxidoreductase, bifunctional alcohol dehydrogenase E (ADHE), acetyl-CoA synthase (ADP forming), and the like [4], or whether they have some other means of surviving without oxygen. It is perhaps more likely that they use strategies more similar to those found in the anaerobic mitochondria of parasitic animals, for example, malate dismutation with the involvement of rhodoquinone [4].

As a long shot alternative, if the animals are alive, it is even imaginable that they have acquired genes via lateral gene transfer (LGT) for a new strategy to survive anoxia. Indeed, some camps argue that all eukaryotes are ancestrally strict aerobes and that the ability of eukaryotes to survive anoxia is always the result of lateral gene transfer [9]. We do not agree with that view, mainly for three reasons. First, the eukaryotic anaerobes studied so far always have the same basic carbon and energy metabolic backbone [4] and if LGT was behind eukaryote anaerobiosis, then eukaryotic anaerobes should be as physiologically diverse as prokaryotic anaerobes, which is definitely not the case; energy metabolism based on sulfate reduction [10], which is lacking in eukaryotes, is a strong case in point. Second, the Earth sciences tell us that anaerobic habitats are ancient and that aerobic habitats are recent [8]. So, if anything, we should be seeing LGT as a means of improving mitochondrial function in aerobic habitats. For example, aerobic methane oxidation is a very widespread form of energy metabolism in prokaryotes but we don’t see eukaryotes that have acquired genes to do that; rather, eukaryotes possess one ancestrally present stock of enzymes [4]. Third, it is often proposed that one lineage of eukaryotes acquires one or the other anaerobic enzyme via LGT from prokaryotes and then passes it around via eukaryote to eukaryote LGT in order to account for the monophyly of the eukaryote enzymes involved. That idea has been specifically tested at the whole-genome level, and rejected, whereby the “patchy gene distributions” that are often seen as the hallmark of LGT are actually better explained by differential loss than they are by LGT [11].

Of course it might also turn out that the loriciferans from the habitats in question do not show vital signs of gene expression. It might be that they are dead, not alive. There is only one way to find out: biologists will have to go back out to those deep environments and get new samples.
